# Sepiolite-Based Adsorbents for the Removal of Potentially Toxic Elements from Water: A Strategic Review for the Case of Environmental Contamination in Hunan, China

**DOI:** 10.3390/ijerph15081653

**Published:** 2018-08-03

**Authors:** Zhenghua Wang, Lina Liao, Andrew Hursthouse, Na Song, Bozhi Ren

**Affiliations:** 1 Hunan Provincial Key Laboratory of Shale Gas Resource Exploitation, Hunan University of Science & Technology, Xiangtan 411201, China; wzh@hnust.edu.cn (Z.W.); xtrbz@sina.com (B.R.); 2 School of Civil Engineering, Hunan University of Science & Technology, Xiangtan 411201, China; liaolina123@foxmail.com; 3 School of Computing, Engineering & Physical Sciences, University of the West of Scotland, Paisley PA1 2BE, UK; na.song@uws.ac.uk

**Keywords:** sepiolite, adsorption, potentially toxic elements, modification, regeneration, Xiangjiang River

## Abstract

The last few decades have seen rapid industrialization and urban development in many regions globally; with associated pollution by potentially toxic elements; which have become a threat to human health and the food chain. This is particularly prevalent in a number of regions in China that host multiple mineral resources and are important agricultural locations. Solutions to protect contamination of the food chain are more effective and sustainable if locally sourced materials are available; and in this context; we review the potential of local (sepiolite) mineral deposits to treat water contamination in the Hunan Municipality; central south China; widely recognized for significant environmental pollution issues (particularly by Hg; Cd; Pb; and Cr) and the high agricultural productivity of the region. Sepiolite is an abundant fibrous clay mineral with modest to good adsorption properties and extensive industrial process applications. It shows reasonable performance as an adsorbent for element removal. In addition; a number of surface modification strategies are available that improve this capability. We review these studies; focused on sorption reaction mechanisms and regeneration potential; with a view to present options for a localized and effective economic strategy for future application.

## 1. Introduction

With the international scale of rapid industrialization and urbanization, many developing and emerging economies have exploited local natural resources. These activities are energy-intensive, associated with significant interventions in the natural ecosystem including the water balance, which leads to emissions of pollutants to water, soil, and air. Metal pollution is one of the most serious and frequently encountered problems. For example, the River Ganga in India, which is considered sacred by Indian society, has serious pollution from Mn, Cr, Pb, Cd, and other potentially toxic elements (PTEs) [1]. Pollutants, such as Cd, Cu and Pb, in the Red River in Vietnam, are also much higher than the local discharge standards, and the maximum enrichment factor for Cd is 19.3 [2]. China’s rapid industrialization has also led to a severe deterioration in water quality in the country’s lakes and rivers. More than 80 percent of Chinese rivers and lakes, including seven key river systems, are contaminated with different types and to different degrees of PTEs with As, Cd, Hg, and Pb being the most frequently detected in these rivers [3,4,5]. Soils in China also suffer from high degrees of contamination. A report of the national survey of soil contamination of China, which was published in 2014, showed that the exceedance of environmental standards for Cd, Hg, As, Cu, Pb, Cr, Zn, and Ni in soil samples reached 7.0%, 1.6%, 2.7%, 2.1%, 1.5%, 1.1%, 0.9%, and 4.8%, respectively [6]. 

The persistence of PTE pollution has the potential to impact on both the human and wider ecological environment because of their long residence time and the potentially toxic impact through biological amplification [7,8]. PTE enrichment in the human body through the food chain eventually destroys the normal function of proteins and enzymes in the body, and high concentrations can form more toxic compounds, which do great harm to organisms [9,10]. Acute toxicity mainly affects the normal function of a particular organ and can damage or destroy the reproductive organs with intergenerational influence on child health [4,11].

Many techniques including adsorption, precipitation, biological treatment, ion exchange, and membrane separation are used to deal directly with PTE pollution [12,13,14]. Among them, adsorption, which refers to the adsorption of metals by means of intermolecular force or electron transfer and electron pair bonding [15], is widely used in pollution control because it is cheap and easy to apply and operate and systems are often reusable. Adsorption processes often refer generically to removal of target pollutants, which may also include other mechanisms such as ion exchange (the exchange of aqueous pollutant ions with available surface ions on the solid phase) and precipitation (where solution conditions exceed solubility conditions for specific species). Many kinds of adsorbent materials have been applied for the removal of soluble pollutant metals such as activated carbon, and modified complex materials such as metal ferrite doped carbon [16], and metal organic framework systems [17]. Capacities can be quite high, for example, 200–300 mg/g for Hg on modified carbon and similar range for, for example, U and Th on metal organic framework materials. However, applications are often limited due to its relatively high synthesis costs. Naturally derived clay minerals like kaolin, zeolite, sepiolite, bentonite, and perlite have also been utilized as alternative low-cost adsorbents for remediation of metal polluted environments. Whilst adsorption capacities for, for example, sepiolite may be an order of magnitude lower than synthetic systems [18] (there are many examples of studies of these materials for the treatment of metals by adsorption that show useful level of performance [19,20]. The focus for the future development of adsorption based systems should be on identifying adsorbent materials that are cheap and effective adsorbents in the context of the treatment scenarios (for example, at point source or to deal with diffuse pollution), to improve the effectiveness of these treatments and to ensure that no secondary pollution is produced [21,22,23,24]. Secondary tasks include the need for good solid/water separation and the regeneration of adsorbent. In this review, we focus on the potential of one mineral system in the context of a local demand for treatment, potentially supported by locally derived materials, which fits with the circular economy principles of resource use and efficiency. There are a number of serious regional pollution problems, within the Central Southern Chinese province of Hunan. It is rich in mineral resources, which have been extensively exploited over recent decades, compromising its significant contribution to food production from a strong agricultural sector. In addition to extensive base metal deposits, one of the regions’ other significant mineral resources is sepiolite, a clay mineral with already widespread industrial and manufacturing applications, located in Xiangtan City, Hunan Province. We focus our review to consider work describing mechanisms for the removal of locally relevant metal pollutants, the modification and regeneration steps from the view of economic efficiency and resource sustainability. We identified a number of published studies of this topic, presented in Chinese literature and academic repositories. Our review, therefore, also provides wider international access to relevant data, which will be of benefit to similar locations worldwide. 

## 2. Metal Pollution in Hunan, China 

**Figure 1:**
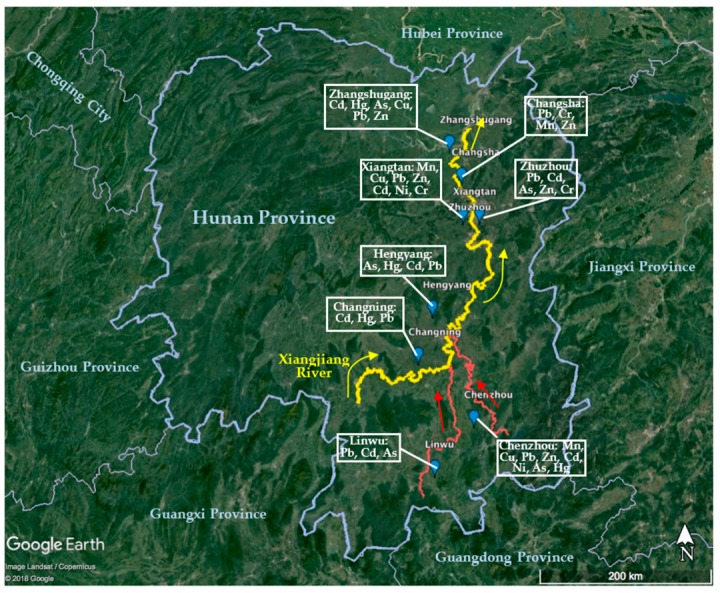
Geographical distribution of the main PTE pollution sources in Xiangjiang Valley. Data from references in Table 1.

It is well known that there are abundant reserves of non-ferrous metals in Hunan Province, and most ores for mining, mineral processing, and smelting of non-ferrous and rare earth elements are located in the Xiangjiang Valley. The Xiangjiang River, which cuts across Hunan Province, is a main water resource for drinking water, process water, and the irrigation of crops. Because of prolonged mining and smelting activities for non-ferrous metals, wastewater has been discharged to the surrounding environment and the Xiangjiang Valley is the most infamous polluted area in central China [25]. Water, soils, and crops in Xiangjiang River basin are heavily contaminated by Cd, Hg, Pb, as well as As [26,27]. The “12th Five-Year” Plan for Comprehensive Prevention and Control of Heavy Metal Pollution indicates that the five major PTE pollutants in China are identified as Pb, Hg, Cd, Cr, and As [28]. In 2015, the state statistics for “the discharge of major pollutants in regional wastewater”, Hg, Cd, and As in Hunan province account for 20.3%, 37.9%, and 32.6% of the total emissions, respectively [29]. The “11th Five-Year Plan” Xiangjiang River basin water pollution prevention and control plan of Hunan province reported that the pollution in Xiangjiang is predominantly caused by Hg, Cd, Pb, As, Zn, and others [30]. The monitoring data for the main pollution indicators in the Xiangjiang are shown in Table 1 with Figure 1 summarizing the geographical distribution of these sources/effects.

## 3. Natural Sepiolite

The mineral sepiolite was discovered by German scholar Woemer in 1789, and the original name of sepiolite *Meerschaum* means “foam of the sea” in German. In 1847, it was officially renamed sepiolite. It is a clay mineral with light color and low density, and has the chemical formula Mg_8_(OH_2_)_4_[Si_6_O_15_]_2_(OH)_4_·8H_2_O [39]. In 1947, sepiolite was first discovered in Jiangxi, China by Chinese geologists Zhang Renjun. By genetic classification, we can divide the sepiolite into two types: sedimentary and hydrothermal sepiolite. The world has proven reserves of around 80 million tons, the main production of raw sepiolite is from deposits in Spain, followed by China, the United States, and Turkey [40]. China has about 30% of the world’s sepiolite reserves. Among Chinese sepiolite reserves, 70% of sepiolite comes from Hunan Province. The city of Xiangtan, in Hunan province, hosts more than 20 million tonnes of sepiolite reserves.

Sepiolite is a clay mineral with a hydrous magnesium silicate, it is a member of the orthorhombic crystal system. It presents a structure of needle-like particles and has talc-like layers that consist of two layers of tetrahedral silica and a central octahedral magnesium layer [41]. As a result of its particular crystal structure, sepiolite has great sorptive, rheological, and catalytic properties, and it is also widely used in a variety of industrial and commercial applications.

The structure of sepiolite is a fibrous needle form, with a hollow channel in the direction of the fiber, which gives special rheological properties. The flow properties of sepiolite means that it is used in drilling muds as a thickener and suspension agent [42,43]. The acidity and alkalinity of sepiolite itself makes its catalytic activity more versatile and widespread. As a negative charge carrier, sepiolite can be utilized to remove pollutant cations [44]. It has a large specific surface area, which can reach 800–900 m^2^/g, and with its porous properties, provides its good access to adsorption sites. This performance can play a part in applications for bleaching, cleaning agent, and other sorption functions. We focus here on those applications relevant to metal adsorption in the context of local environmental contamination. 

## 4. Modification of Sepiolite 

Despite the wide application of sepiolite in a variety of industrial processes when compared with other sorbent systems, it has relatively low surface acidity, narrow channels, low surface area, and poor thermal stability. This limits some applications of natural sepiolite [44]. The adsorption performance of the “modified” sepiolite can be much better than that of natural sepiolite, and studies have shown that the specific surface area can be increased significantly from 29–87 m^2^/g [45]. The adsorption and removal capability of magnetic modified sepiolite for the heavy metal Cr (VI) is 10 times that of natural sepiolite [46] and of similar magnitude in the case of Hg^2+^ for surfactant modified sepiolite [18], but is still 0.5 to 0.3 of modified carbon and synthetic metal organic systems highlighted in the introduction above. When comparing natural sepiolite with a number of modified sepiolite systems to remove Pb^2+^, it was found that the order of adsorption capacity is as follows: H_2_O_2_ modified sepiolite > KNO_3_ modified sepiolite > natural sepiolite. When the initial Pb^2+^ concentration is 2.5 mg/L, the adsorption capacity of H_2_O_2_ modified sepiolite is twice as much as natural sepiolite [47]. According to other studies, the adsorption of Cr (VI) by activated sepiolite follows the following order: acid activated-mercapto silane organic modified sepiolite > sulphur silane modified sepiolite > acid activated modified sepiolite > natural sepiolite [48]. For the adsorption of Pb^2+^ thermally modified sepiolite > natural sepiolite > and for the adsorption of Cd^2+^ thermally modified sepiolite > natural sepiolite [49]. This provides a range of strategies for modification and sources of reactants to enable optimization of technological approach.

### 4.1. Acid Treatment 

In acid treatment the reaction with carbonate in the sepiolite dissolves these impurities and clears any surface obstruction. The H^+^ from the acid will replace the Ca^2+^, Mg^2+^, Na^+^, and K^+^ in the sepiolite interlayer, and improves the access to the surface and cavities in the sepiolite and increases the surface area and microporosity to provide improved adsorption performance [48,50,51].

### 4.2. Thermal Treatment 

Thermal modification of sepiolite is the process of calcining natural sepiolite at different temperatures. At the different temperatures, the associated water in hygroscopic, zeolitic, and coordinated and structural octahedral hydroxyls groups will be removed to reduce the water film resistance, increasing the porosity and in doing so, improving the adsorption performance of sepiolite [52,53,54].

In calcination temperatures around 500 °C, the adsorbed water disappears from the sepiolite structure and apertures expand, with magnesium loss from the mineral structure. This increases the metal ion space, which is beneficial for the removal of Cr^3+^, at the same time. The removal ability of heavy metals from wastewater was improved because of the change of internal cavity structure of the thermally treated sepiolite [55].

### 4.3. Magnetic Modification 

After treatment of heavy metal contaminated wastewater by sepiolite, it is difficult to achieve good separation of mineral/water mixtures, which leads to the difficulty of secondary reuse of sepiolite. The magnetization of sepiolite provides an effective way to facilitate separation and allows further treatment and/or reuse. In addition to using iron-based systems, the Fe^3+^ present in the modified sepiolite has oxidative properties, and the dissolution of Fe^3+^ adds acidity, which is beneficial to the removal of heavy metal ions [46,56,57,58]. In the process of adsorption, the phase structure of magnetic sepiolite did not show any obvious change. The adsorption mechanism is an ion exchange process between heavy metal ions and zeolite in the magnetised sepiolite crystal and coupled adsorption occurs between the heavy metal ions and the hydroxyl groups (Fe–OH and Si–OH) on the surface of magnetised sepiolite [59].

### 4.4. Organic Modification

Organic modification uses a range of molecules such as surfactants, polymerised organic matter, or microorganisms to load or graft copolymerization to the surface or in a cavity of the sepiolite and modify its structure and the properties of the material surface. This is a relatively new area of research for metal removal from wastewater [48,60,61,62,63].

### 4.5. Acid Thermal Treatment 

Acid thermal modification combines acid treatment with thermal modification of sepiolite. This approach to removing metal ions from water is widely used, and the adsorption using this acid thermal modified sepiolite is much better than for natural sepiolite, or separate acid modified and thermal treatment sepiolite. The sepiolite was treated with hydrochloric acid solution, and then calcinated at 450 °C, and subsequently used to treat Zn^2+^, Pb^2+^, Cu^2+^, and Cd^2+^ in solution through an ion exchange and surface complex adsorption process [48].

## 5. Examples of the Application of Sepiolite to Potentially Toxic Element Removals from Aqueous Environmental Systems

It has been reported that sepiolite-based materials can be used to remove a wide range of pollutant elements from water and soils. We focus on reports relating to the key pollution metal elements in Hunan province (Hg, Cd, Pb, and Cr), where most of the research is still at the bench-scale phase.

### 5.1. The Removal Hg^2+^ from Wastewater

**Figure 2:**
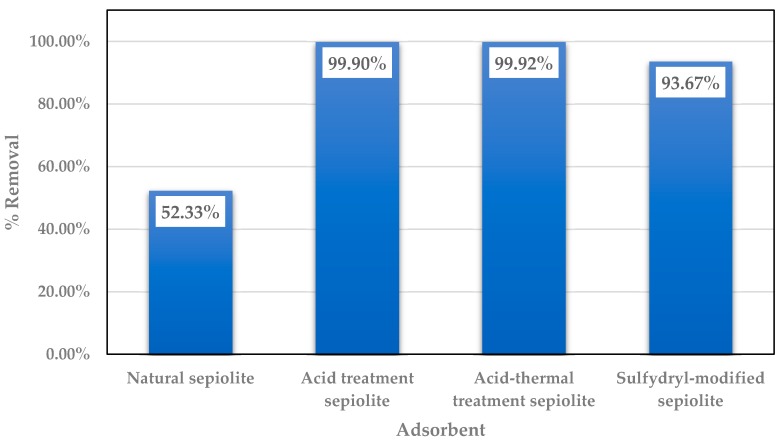
The removal of Hg by sepiolite and various modified sepiolite products [64,65,66].

There are few studies on the removal of mercury ions using sepiolite and modified sepiolite. Those that are available show that modified sepiolite has a significantly improved effect on Hg^2+^ removal. As shown in Figure 2, the removal of Hg^2+^ was more than 90% after acid modification, acid thermal modification, and organic modification, compared with the natural sepiolite (about 50%).

The mechanism of removing Hg^2+^ using sepiolite and different modified conditions is also different. The reaction mechanism for acid modified sepiolite is the dissolution of the impurities in the sepiolite by the acid, at the same time, the pore area is enlarged, and the acidic hydroxyl group is exposed to contact with the Hg ions [65]. In the case of acid thermal treatment, the mechanism of the reaction with adsorbing ions is mainly due to the combination the approach for both acid and thermal activation. The process of dissolving the impurities in the sepiolite results in improved thermal removal of the structural water in the sepiolite and contact resistance is reduced. The combined effect is better than that of pure acid modification or thermal modification [66]. The reaction of sulfhydryl modified sepiolite for Hg^2+^ conforms to the pseudo-second-order kinetics equation, and adsorption of Hg^2+^ onto sepiolite fits Langmuir and Freundlich isotherm models. The regression coefficient (R^2^ = 0.994) suggests that Hg^2+^ adsorption on sepiolite more closely followed the Langmuir model. The sulfhydryl modified sepiolite has a smoother surface, and its internal pores are enlarged, and the increased negative charge is conducive for reaction with metal ions, so as to more effectively remove the Hg^2+^ [64].

### 5.2. The Removal of Cd from Wastewater 

**Figure 3:**
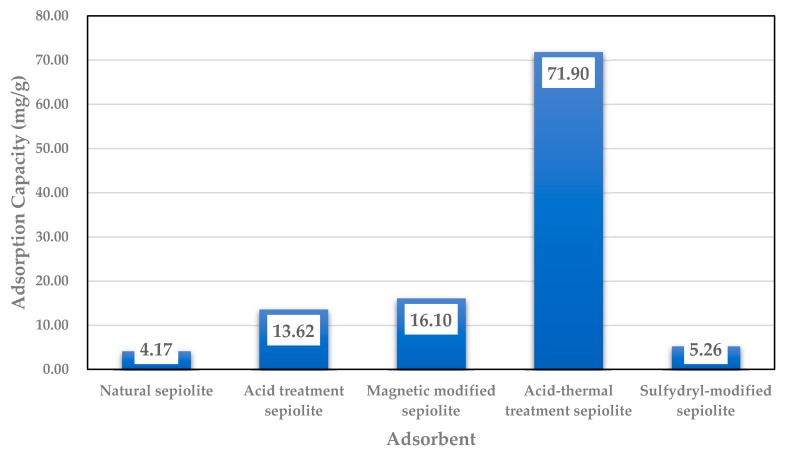
Adsorption capacity of Cd by sepiolite from solutions [66,68,70,71].

Studies found that the adsorption reaction of sepiolite with Cd^2+^ conforms to a pseudo-second-order kinetics model, and its R^2^ is 0.999. It also satisfies the isothermal adsorption model of both Langmuir and Freundlich, and the degree of fit to the Langmuir isothermal model is high (R^2^ = 0.999). The saturated adsorption capacity of sepiolite for Cd^2+^ was 11.48 mg/g, and the saturated adsorption capacity of the acid modified sepiolite for Cd^2+^ was 13.62 mg/g [67,68]. The study on the adsorption of heavy metal Cd by sepiolite on acid and thermal treatment found that the adsorption of Cd^2+^ was increased by calcining the sepiolite, the main reason is that the CaO produced by the high temperature roasting makes the liquid alkaline, so that the Cd^2+^ was removed by precipitation reactions, in the process of acid treatment, the treatment effect of sulfuric acid is better than that of nitric acid and hydrochloric acid, which is due to the precipitation reaction between H_2_SO_4_ and Cd^2+^ [69]. The reaction mechanism of magnetic modified sepiolite to treat Cd^2+^ showed that the degree of fit to the Langmuir isothermal model was higher than Freundlich model, which indicates that the adsorption reaction of magnetic modified sepiolite and Cd^2+^ was based on single ion layer surface coverage [70]. Figure 3 shows that the adsorption capacity of Cd^2+^ was increased 3–17 times after modification, especially for the combined acid thermal modification.

### 5.3. The Removal of Pb from Wastewater

The adsorption of Pb^2+^ with sepiolite and modified sepiolite departs from the Langmuir isothermal model, as a result of the precipitation caused by the reaction. The reaction mechanism is not only due in part to the complexation at the ion exchange surface, but also that the Pb^2+^ will precipitate during the reaction process [68]. The reaction mechanism of sulfhydryl modified sepiolite and Pb^2+^ fits well with the Freundlich isothermal model, and it also conforms to the pseudo-second-order kinetics, for which the R^2^ = 0.9976, and its maximum adsorption capacity for Pb is 97 mg/g [63]. The adsorption of Pb by natural sepiolite and iron oxide-coated sepiolite was found that both fit well with the Freundlich and Langmuir isothermal models, with the degree of fit for the Langmuir model was the best (R^2^ = 0.990). It can be seen that the reaction mechanism of sulfhydryl modified sepiolite and natural sepiolite and iron oxide coated sepiolite differ. The introduction of sulfhydryl group and the stable coordination bond with the heavy metal ions in the sulfhydryl group have a good influence on adsorption, the adsorption effect of iron coating sepiolite on pollutant metals is higher than that for natural sepiolite, which may be due to replacement of the zeolite water in the sepiolite and increase in adsorption sites [72,73]. In addition, the effect of organic modification (dodecyl benzene sulfonic acid sodium and sodium chloride modified sepiolite) on the adsorption of Pb^2+^ is greater than that of unmodified sepiolite and its mechanism is related to the organic modification providing surface enrichment of macromolecule groups for the adsorption of metal ions. Adsorption isotherm has a good fit to the Langmuir model and pseudo-second-order kinetic equation, the quasi secondary maximum adsorption capacity is 226.8 mg/g [74]. The sepiolite has a good performance for the adsorption of Pb^2+^ after acid soaking and high temperature calcination, and its removal at 50 mg/L Pb^2+^ is 80.9%. The reaction has a best fit to the Freundlich adsorption isotherm [75].

### 5.4. The Application of Remove Cr in the Wastewater by Sepiolite

The mechanism for sepiolite adsorption of Cr (VI) occurs in two stages. Firstly, to remove part of the contamination by surface of physical adsorption, followed by the likely reduction of Cr (VI) to Cr (III) [76,77].

At pH = 2, it was found that amine functionalized natural and acid-activated sepiolites [78] had the best adsorption effect on Cr (VI), and the adsorption capacity was 37 mg/g and 60 mg/g, respectively. The system showed a good fit to the Freundlich isothermal model compared with Langmuir and D–R isothermal models. It shows that in the process of adsorption should be simultaneously to multiple sites. By analyzing the R^2^ value for the pseudo-second-order kinetics, the cause of limited adsorption efficiency in chemical adsorption process can be analyzed. In the study of the adsorption treatment of Cr (VI) with sepiolite-supported nanoscale zero-based iron (S-NZVI), the maximum adsorption capacity of S-NZVI for Cr (VI) was 43.86 mg/g, with its response fitting well to the Freundlich isothermal model, the R^2^ is greater than for Langmuir isothermal model, which suggested that that is due to the S-NZVI surface heterogeneity the effects on the Cr (VI) removal, the linear relationship between the removal of Cr (VI) and the input of S-NZVI fits to pseudo-first-order kinetics [77]. In the study of the adsorption of Cr on magnetic modified sepiolite, it was found that the reaction is also strongly related to the Freundlich isothermal model. This shows that the magnetic surface of modified sepiolite exhibits heterogeneity, with adsorption between monolayer and multilayer adsorption mechanisms. The reaction has a good fit to pseudo-second-order dynamics (R^2^ > 0.99) and the maximum adsorption capacity was 3.6 mg/g. Compared with natural sepiolite, the removal of Cr (VI) by modified sepiolite is much higher than for natural sepiolite [46]. 

Other studies of natural sepiolite adsorption of Cr^3+^, Cd^2+^, and Mn^2+^ showed the best adsorption effect for Cr^3+^ was on natural sepiolite, with good fit to the Langmuir isothermal model. The adsorption process is not only the ion exchange, but also the formation of complex and surface adsorption [79].

## 6. Regeneration of Sepiolite

The process of regeneration of modified sepiolite treated with wastewater is to restore most of the adsorption capacity of sepiolite, so that the material can be reused, reducing operational costs and preserving resources, which fitswith green production process philosophy. At present, there are few reports on the adsorption and regeneration of sepiolite, and it is of great significance to find an economical and efficient method for the treatment of waste water [80,81].

The method of sepiolite regeneration includes: acid regeneration, alkaline regeneration, and salt regeneration. Jia et al. used salt regeneration to regenerate the sepiolite that had adsorbed Zn^2+^, it shows that the capacity adsorption of regenerated sepiolite is still high although some minor reduction occurs [82]. The adsorption ability of sepiolite pre- and post-regeneration is shown in Table 2.

From Table 2, we can see that the regenerated sepiolite maintains good adsorption properties for metals ions. Li et al. [87] used hydrochloric acid, sodium chloride, and sodium hydroxide in three ways to regenerate the sepiolite. It was found that NaOH had the best treatment effect on the regeneration of sepiolite, and the treatment effect was similar with that of water and sodium chloride, with the use of hydrochloric acid being the poorest. Li et al. [88] used two different kinds of acid, salt and alkaline, to study sepiolite regeneration, and showed that the NaOH and nitric acid had the best effect on sepiolite regeneration, but hydrochloric acid and NaOH treatments were not ideal for sepiolite re-use. Yan showed several different methods for the regeneration of sepiolite: two acid regeneration (HNO_3_, HCl), alkaline regeneration, and salt regeneration. It shows that the removal of metal ions was reduced by 19.72% when the regenerating solution is HNO_3_, the removal of metal ions was reduced by 16.28% when the regenerating solution is HCl, for NaCl, it was reduced by 5.32% and after five steps, for NaOH, the removal of metal ions was reduced by 6.24% [89]. The results of these trials show that regeneration process is metal specific, which must be considered when developing protocols for full trials and field applications.

## 7. Conclusions

Pollution from mining and industry continues to be serious environmental problem in Xiangjiang Valley, which is the key rice production area in Hunan province. We need to identify approaches to deal with the protection of the food chain over widespread areas. Adsorption is still an effective technology for removing metals from water, but requires careful consideration to deal with multiple metals, sources, and regional scale contamination. In this review, sepiolite based materials were selected as target adsorbents for heavy metals removal because of their local abundance and potential cost effective application. The characteristics and function of natural and modified sepiolites are reviewed and compared. The sorption performance of modified sepiolite obtained from acid, magnetic, organic, and thermal treatment is significantly improved over natural materials and can be re-used through regeneration. These methods include acid, alkaline, and salt regeneration. The sorption performance of sepiolite after regeneration is greater than 70% of its original performance.

We identified that the relatively low efficiency for heavy metal removal by natural sepiolite was because of the scarcity of exchange sites for contaminant metal ions and capacities are much lower than other synthetic sorbent materials. However, the modification of sepiolite is viable and provides potentially useful adsorption capacity. However, the process of modification also increases the cost of any potential application. In our review, few reports have considered the cost–benefit for modification of sepiolite and application of modified sepiolite on removing metals. Most studies still focus on the mechanism of modification and regeneration of sepiolite. In order to fully establish the potential of sepiolite, as a low-cost and effective adsorbent, further field scale research involving a product life cycle approach is required. This will identify the full potential of local resources to treat local pollution and meet with recent national (China) international (EU–China) agreements on resource conservation and environmental protection.

## Figures and Tables

**Table 1:** A summary of monitoring data for pollution indicators in Xiangjiang River.

Monitoring Locations in Xiangjiang River	PTE (s)	Pollution Condition	Reference
Upstream, Chenzhou Reach	Mn, Cu, Pb, Zn, Cd, Ni, As, Hg	Most of the elements exceed the standard e.g., Cd exceeds the discharge standard by about 1.2–9 times.	[31]
Gan river, Linwu (A tributary of Xiangjiang River)	Pb, Cd, As	Compared with the limit value of Environmental quality standards for surface water, Pb exceeds 109 times, Cd exceeds 242 times, As exceeds 75.8 times.	[32]
Changning Reach	Cd, Hg, Pb	Cd, Hg, and Pb were above the limit of the emission standard 265, 104.2, and 13.8 times, respectively.	[31]
Hengyang Reach	As, Hg, Pb, Cd	Compared with the limit value of Environmental quality standards for surface water, As, Hg, Pb, and Cd exceed the standard; 13.58%, 8.94%, 2.32%, and 27.16%, respectively.	[33]
Hengyang–Changsha Reach	As, Cd, Pb, Zn, Cu	Compared with the China Environmental Quality Standard for Soil Metals (GB15618-1995, Grade II), in the sample sites, the ratio of exceedance for As, Cd, and Pb was 13.2%, 68.5%, and 8.7% of soil samples, respectively. Cd, Pb, Zn, and Cu were much higher than their respective background values in the soil of Hunan Province, being 83.1–1178.7, 4.46–15.9, 2.88–16.1, and 3.35–6.22 times as high, respectively.	[34,35]
Changsha–Zhuzhou–Xiangtan Reach	Mn, Zn, Pb, Cu, Cr, Ni	Serious pollution of Pb and Zn, mild or moderate pollution of Mn and Cu in Xiawan, Zhuzhou Reach; moderate pollution of Pb and Zn, mild or moderate pollution of Cu, Mn, Ni, and Cr in Xiangtan and Changsha Reach.	[36]
Zhuzhou Reach	Cd, As, Pb, Zn, Cr	Cd in tobacco leaves is 6.98–37 mg/kg, Cd in cabbage is 15.4–18.3 mg/kg, Cd in rice is 1.03–1.78 mg/kg, Cd in amaranth is 6.03 mg/kg. As levels in vegetables are five times higher than normal.	[31,36]
Xiangtan Reach (manganese mine)	Mn, Cu, Pb, Zn, Cd, Ni	The average contents of Mn, Cu, Pb, Zn, Cd, and Ni are as follows: 7990.21 mg/kg, 66.38 mg/kg, 401.15 mg/kg, 640.32 mg/kg, 13.15 mg/kg, and 91.33 mg/kg. Their content is more than the national average worth several times or even dozens of times.	[37]
Entrance of Dongting Lake	Cd, Hg, As, Cu, Pb, Zn	Cd, Hg, As, Cu, Pb, and Zn in the sediments were 3.27, 0.190, 27.10, 39.8, 38.0, and 157.8 mg /kg, respectively.	[38]

**Table 2:** The adsorption ability of sepiolite before and after regeneration.

Regeneration Methods	Metal (s)	Before Regeneration	After Regeneration	Reference
HCl	Ga^3+^	The removal is 98.8%	The removal is 94.4% after the fourth cycle of adsorption–desorption	[83]
	Pb^2+^	equilibrium absorption capacity is 638.9 mg/g	The equilibrium absorption capacity is 489.2 mg/g after fifth cycle of adsorption–desorption	[84]
HNO_3_	Pb^2+^	The saturated adsorption capacity is 114.2 mg/g	The saturated adsorption capacity is 97.6 mg/g	[66]
	Hg^2+^	The saturated adsorption capacity is 84.6 mg/g	The saturated adsorption capacity is 64.1 mg/g	[66]
	Cd^2+^	The saturated adsorption capacity is 71.9 mg/g	The saturated adsorption capacity is 52.5 mg/g	[66]
	Fe^3+^	/	Removal of iron ions was decreased less than 5% after the fourth cycle of adsorption-desorption	[85]
NaOH	Co^2+^	The sorption capacities of Co^2+^ is 16.02 mg/g,	The sorption capacities of Co^2+^ is 14.50 mg/g after the sixth cycle of adsorption-desorption	[86]
	Cd^2+^	The sorption capacities of Cd^2+^ is 12.38 mg/g	The sorption capacities of Cd^2+^ is 10.99 mg/g after the sixth cycle of adsorption–desorption	[86]
	Sb	/	Removal efficiency was decreased less than 7% after the fifth cycle of adsorption–desorption	[87]
NaCl	Pb, Zn	/	Removal efficiency was decreased by 21.27% after the fifth cycle of adsorption–desorption	[88]
